# The Bostrichidae of the Maltese Islands (Coleoptera)

**DOI:** 10.3897/zookeys.481.8294

**Published:** 2015-02-04

**Authors:** Gianluca Nardi, David Mifsud

**Affiliations:** 1 Centro Nazionale per lo Studio e la Conservazione della Biodiversità Forestale “Bosco Fontana”, Sede di Bosco Fontana – Corpo Forestale dello Stato, Strada Mantova 29, I-46045 Marmirolo (MN), Italy; 2 Institute of Earth Systems, Division of Rural Sciences and Food Systems, University of Malta, Msida MSD 2080, Malta

**Keywords:** Bostrichidae, new records, new synonym, alien species, Malta, Italy

## Abstract

The Bostrichidae of the Maltese Islands are reviewed. Ten species are recorded with certainty from this Archipelago, of which 6 namely, *Trogoxylon impressum* (Comolli, 1837), *Amphicerus bimaculatus* (A.G. Olivier, 1790), *Heterobostrychus aequalis* (Waterhouse, 1884), *Sinoxylon unidentatum* (Fabricius, 1801), *Xyloperthella picea* (A.G. Olivier, 1790) and *Apate monachus* Fabricius, 1775 are recorded for the first time. Two of the mentioned species (*Heterobostrychus aequalis* and *Sinoxylon unidentatum*) are alien and recorded only on the basis of single captures and the possible establishment of these species is discussed. Earlier records of *Scobicia pustulata* (Fabricius, 1801) from Malta are incorrect and should be attributed to *Scobicia chevrieri* (A. Villa & J.B. Villa, 1835). A zoogeographical analysis and an updated checklist of the 12 species of Bostrichidae recorded from the Maltese Islands and neigbouring Sicilian islands (Pantelleria, Linosa and Lampedusa) are also provided.

Rhizopertha dominica (Fabricius, 1792) form granulipennis Lesne in Beeson & Bhatia, 1937 from Uttarakhand (northern India) was overlooked by almost all subsequent authors. Its history is summarized and the following new synonymy is established: Rhizopertha dominica (Fabricius, 1792) form granulipennis Lesne in Beeson & Bhatia, 1937 = *Rhyzopertha dominica* (Fabricius, 1792), **syn. n.**

Finally, records of *Amphicerus bimaculatus* from Azerbaijan, of *Bostrichus capucinus* (Linnaeus, 1758) from Jordan and Syria, of *Scobicia chevrieri* from Jordan and Italy, of *Xyloperthella picea* from Italy, and of *Apate monachus* from Corsica (France) and Italy, are also provided.

## Introduction

The larvae of most species of this family are wood borers, and as other saproxylic organisms they play an important role in the decomposition processes. They are thus significant for nutrient cycling in natural and semi-natural ecosystems, particularly forests (cf. Stokland et al. 2012). Several species of this family are of great economic importance since they can cause extensive damage to dry and dead wood, to seasoned sapwood timber, to bamboo, and to wooden or bamboo artifacts through the boring behavior of both adults and larvae. Moreover, several other species are pests of living trees and vines, a few species are store product pests attacking commodities such as grain and tubers. These beetles are frequently transported between countries, especially in wood packing materials such as crating and dunnage, and are often intercepted at ports and cargo distribution centres (cf. Haack and Slansky 1987, Geis 2002, Ivie 2002, Haack 2006, Liu et al. 2006, Bahillo de la Puebla et al. 2007, Liu et al. 2008, Lawrence 2010).

The earliest record of the family comes from the mid Cretaceous (Peris et al. 2014). This family is now mainly represented in subtropical and tropical regions, and currently about 600 described species accommodated in 90 genera are known to occur worldwide (cf. Lawrence and Slipinski 2013, Zahradník and Háva 2014).

The knowledge on Maltese Bostrichidae was very limited, with only five previously recorded species. New data on Maltese Bostrichidae emerged from recent studies carried out on collections made from the Maltese Islands, the results of which are included in the present work.

## Material and methods

### Study area

The study area comprises all of the Maltese islands (c. 316 km²). Chetcuti et al. (1992), Schembri (1993, 1997) and Giusti et al. (1995) can be consulted for general environmental information on this Archipelago situated in the centre of the Mediterranean basin.

### Nomenclature and classification

The suprageneric classification of the family and the nomenclature (family-group names and genus-group names) adopted in the present work follow Zahradník and Háva (2014), but the subfamilies follow Liu and Schönitzer (2011). The species are listed alphabetically as in Borowski and Węgrzynowicz (2007) and Ivie (2010).

### Faunistic list

For each species, the following information is provided: nomenclatural combinations (listed chronologically-alphabetically) of the Maltese records found in the literature, literature records on the Maltese Islands, material examined, chorotype, data on ecology, and notes.

When possible, the following data was also provided for each record: island, municipality, locality of collection, altitude, date of collection, collector/s, collecting method, number of specimens, and, in parenthesis, literature reference or abbreviation of the depository. The records are listed in alphabetic order with respect to localities of collection. A semicolon separates different records; if these are from the same site, the name of the site is listed only at the beginning with the older record. When deemed useful for the discussion of some species, material examined from other countries (“Other material examined”) is also provided.

Possible interpolations are given in square brackets; collecting data of Italian specimens that is originally written in Italian, is hereunder provided in English. Information on very old specimens is written in double quotation marks.

Regarding literature records, Luigioni (1929) listed the two previously recorded bostrichids from Malta (cf. Cameron and Caruana Gatto 1907), but both Porta (1929, 1934, 1949, 1959) and Borowski (2007) overlooked these records. Records from “Malta” of Nardi (2004a, 2004b) are based only on previous literature. Finally, Schembri and Lanfranco (1996: 6) recorded an undentified bostrichid collected in a consignment of tropical logs imported from Africa, but the material was presumably lost (D. Mifsud, unpublished data).

The identifications of the material examined were made by one of us (G. Nardi), and were based on Lesne (1899, 1901b, 1904, 1906, 1909, 1924), Español (1955, 1956a), Cymorek (1961), Liu et al. (2006), Bahillo de la Puebla et al. (2007), Sitticaya et al. (2009) and Beiriger (2010). References, including illustrations of the genitalia are listed when available. This was done since so far little attention was given to the study of the genitalia of these beetles.

### Zoogeography

Chorotypes, which were also used in the zoogeographical analysis, were assigned according to Vigna Taglianti et al. (1993, 1999) and are based on the distributions provided by the authors cited for each species. Moreover, the chorotypes of many species show extensions or more confined distributions when compared to the standard ones; these differences were mentioned only when the change is significant. For further information on global distributions, references cited in the text may be consulted.

### Acronyms

**Specimen depositories**

BMNH The Natural History Museum, London, UK;

CCI private collection P. Cornacchia, Porto Mantovano (Mantua), Italy;

CMM private collection D. Mifsud, Malta;

CNBFVR Centro Nazionale per lo Studio e la Conservazione della Biodiversità Forestale “Bosco Fontana” di Verona. Sede di Bosco Fontana. Marmirolo (Mantua), Italy;

CNI private collection G. Nardi, Cisterna di Latina (Latina), Italy;

MCSV Museo civico di Storia Naturale, Verona, Italy;

MCSVA F. Angelini collection c/o Museo civico di Storia Naturale, Verona, Italy;

MCSVD G. Dellabeffa collection c/o Museo civico di Storia Naturale, Verona, Italy;

MCZRD E. De Maggi collection c/o Museo civico di Zoologia, Rome, Italy;

MCZRL P. Luigioni collection c/o Museo civico di Zoologia, Rome, Italy;

MZUF Museo di Storia Naturale dell’Università degli Studi di Firenze, Sezione di Zoologia “La Specola”, Florence, Italy;

MZUR Museo di Zoologia, Università degli Studi di Roma “Sapienza”, Rome, Italy.

**Main collectors**

AF A. Falzon;

DD D. Dandria;

D.P.I.T. Dana Project Invertebrate Team = [S. De Felici, T. Di Micco De Santo, M. Shubat, F. Al-Eman Al-Husseini & A. Abu Hantash];

DM D. Mifsud;

GN G. Nardi;

HB H. Borg Barthet;

LC L.F. Cassar;

LF L. Fancello;

PS P. Sammut.

**Other abbreviations**

ex = specimen/s;

Fraz. = Frazione = Hamlet;

prov. = province;

pt = pitfall trap.

## Results

### Subfamily Lyctinae Billberg, 1820, Tribe Lyctini Billberg, 1820

#### Lyctus brunneus

Taxon classificationAnimaliaColeopteraBostrichidae

(Stephens, 1830)

Lyctus brunneus Steph.: Cameron and Caruana Gatto 1907: 398.
Lyctus (Xylotrogus) brunneus Steph.: Luigioni 1929: 642.
Lyctus brunneus (Stephens, 1830): Nardi 2004a, Denux and Zagatti 2010: 366.

##### Literature records.

**Malta:** Valletta (Cameron and Caruana Gatto 1907); “Mal.” [= Malta] (Luigioni 1929); Malta (Nardi 2004a, Denux and Zagatti 2010).

##### Material examined.

**Malta:** Naxxar, 7.IX.1997, DD, [in human abitation], 2 ♂♂ (CMM); Rabat, Wied ta l-Isqof, 28.VI.2002, PS, [in an agricultural area], 1 ♀ (CNI).

##### Chorotype.

Cosmopolitan (Borowski 2007, as Lyctus (Xylotrogus) brunneus). This species is widespread in the Palaeartic region (cf. Nardi 2004a, Borowski 2007), but the followings countries were overlooked in the species distribution summarized by Nardi (2004a) and/or Borowski (2007): Algeria (Peyerimhoff 1919, as Lyctus (Xylotrogus) brunneus, Lesne 1924), Austria (Horion 1961, Lucht 1987, Adlbauer 1998, Nardi 2004a, Denux and Zagatti 2010, Querner et al. 2011), Azores (cf. Borges 1990, Nardi 2004a, Oromí et al. 2010), Belarus, Bulgaria (Denux and Zagatti 2010), Canary Islands (cf. Machado and Oromí 2000, Nardi 2004a, Oromí et al. 2009), Corsica (cf. Becker 1969, Geis 2002), Croatia (Damoiseau 1966, Nardi 2004a), Cyprus (Baudi di Selve 1873, 1874, Georghiou 1977, Nardi 2004a), Egypt (Kaszab 1959, Attia and Kamel 1965, in both cases as Lyctus (Xylotrogus) brunneus, Hanna 1970, Alfieri 1976, Hamad and Aly 1985), Fujian (southeastern China) (Vrydagh 1960), Greece (Damoiseau 1966, Nardi 2004a, Denux and Zagatti 2010), Iran (Adeli 1972, Niloufari 1985), Israel (cf. Halperin and Geis 1999, Chikatunov et al. 2004b, 2006), Latvia (Denux and Zagatti 2010), Poland (cf. Burakowsi et al. 1986, Nardi 2004a, Jabłoński et al. 2007, Krajewski and Mazurek 2009), Serbia and Montenegro (Glavendekic et al. 2005, Denux and Zagatti 2010) and Turkey (cf. Gerberg 1957, Akbulut et al. 2008).

##### Ecology.

In tropical areas this species develops in the wood of a large number of unrelated plants, whereas in temperate regions (where the species is considered as an established alien) it develops mainly in hardwood timber (e.g. *Castanea sativa*, *Fraxinus exelsior*, *Junglans regia*, *Quercus* spp., *Ulmus* spp., etc.) primarily in synanthropical environments (workshops, plywood industries, private houses). In the West Palaearctic region, the species was also found in local trees (*Alnus*, *Eucalyptus*, *Ulmus*, etc.) which were in poor health conditions. The passive transport of this beetle has been documented with both wooden products (timbers, tables, furniture, ethnographic collection, briers, etc.) and manioc (cf. Lesne 1924, Lepesme 1944, Español 1956a, Gerberg 1957, Cymorek 1961, Burlini 1972, Aitken 1975, Cavalloro and Ratti 1978, Gambetta and Orlandi 1982a, Gambetta 1983, Wang et al. 1996, Halperin and Geis 1999, as Lyctus (Xylotrogus) brunneus, Geis 2002, Peters et al. 2002, Chikatunov et al. 2004, Bahillo de la Puebla et al. 2007, as Lyctus (Xylotrogus) brunneus, Mattson et al. 2007, Krajewski and Mazurek 2009, Denux and Zagatti 2010, Geis 2012). According to Gambetta and Orlandi (1982b: 55), *Lyctus brunneus* and *Trogoxylon impressum* (Comolli, 1837) are the two species of Lyctinae which are the most widespread in woods in Italian storage situations.

##### Notes.

A species native to Asia which has been established throughout Europe for more than 150 years (Denux and Zagatti 2010). The recently collected material confirm its presence in Malta. In the nearby countries, the species is recorded from Tunisia (Lesne 1924, Normand 1936, Borowski 2007) but not from Sicily (cf. Audisio et al. 1995, Sparacio 1997, Chiappini et al. 2001, Nardi 2004a, Denux and Zagatti 2010). Accurate information and illustrations on the morphology of all stages of this species were provided by Iwata and Nishimoto (1981, 1982) and Kucerová and Stesjkal (2008). Moreover the genitalia of both sexes was illustrated by different authors (e.g. Altson 1924: pls. 31–34, Gerberg 1957: pl. II, figs 15–16, Cymorek 1961: 81, fig. 3c, Iwata and Nishimoto 1982: 19, figs 42–44).

### Subfamily Lyctinae Billberg, 1820, Tribe Trogoxylini Lesne, 1921

#### Trogoxylon impressum

Taxon classificationAnimaliaColeopteraBostrichidae

(Comolli, 1837)

##### Material examined.

**Malta:** Busket, 15.VI.2003, DM, 1 ex (CMM); Mistra Valley, 21.IV.1994, DM, under leves of *Ficus carica*, 1 ex (CMM); Qormi, Ħal-Farrug, 5.V.2003, DM, [on] *Ceratonia siliqua*, 1 ex (CNI); 5.V.2003, DM, 2 ex (CMM; CNI); Rabat, Ta Koronja, 14.VI.2002, PS, 1 ex (CMM); Wied Badu, 3.VII.2002, DM, 1 ex (CMM).

##### Chorotype.

Turanic-European-Mediterranean. This species is widespread from the Canary Islands to Turkmenistan; to the north it reaches Austria, Czech Republic, Germany, Hungary, Slovakia and Switzerland, moreover it has been introduced in some northern European states (Denmark, Finland, Norway and Sweden), into Argentina, Chile and USA (cf. Borowski 2007, Borowski and Węgrzynowicz 2007, Barriga and Cepeda 2009), and it is considered as an established alien in the latter two mentioned territories (Gerberg 1957). The species is possibly also establishment in China (cf. Peters et al. 2002), South Africa and Australia (Geis 2002), but for these territories this was not confirmed (Borowski and Węgrzynowicz 2007). This species was also recorded from Iraq (Knopt 1972) and was introduced to Benelux (Lucht 1987, Drumont and Grootaert 2011) and both countries must be added to its distribution which was summarized by Borowski (2007). Hopkins (1911: 137, as *Lyctus impressus*) in an Appendix to Kraus (1911), recorded this species also for “Cordova, Mex. [= Mexico]”; this record was overlooked since Lesne (1938) probably because this Appendix, which also included data on additional specimens, was added after the submission of the paper of Kraus (1911) (cf. Hopkins 1911: 130, footnote) and Mexico was not included in the mentioned work.

##### Ecology.

*Trogoxylon impressum* is a polyphagous species; in the Mediterranean area it has been reared from many local and exotic trees and shrubs (e.g. *Ceratonia siliqua*, *Cercis siliquastrum*, *Eucalyptus* spp., *Ficus carica*, *Pistacia lentiscus*, *Pistacia vera*, *Punica granatum*, *Quercus* spp., *Tamarix* sp., *Vitis vinifera*), and also from timber (cf. Nardi and Ratti 1995, Halperin and Geis 1999, Geis 2002, Liberto and Audisio 2005, Bahillo et al. 2007, Baena and Zuzarte 2013).

##### Notes.

This species is a new record for the Maltese Islands; it is recorded also from the nearby Island of Pantelleria (Nardi and Ratti 1995) and from the Balearic Islands (cf. Schaufuss 1882, as *Lyctus impressus capitalis* n. var., Bahillo de la Puebla and López-Colón 2001, Nardi 2004a).

Its male genitalia was figured by Gerberg (1957: pl. XI, figs 15–16) and Iablokoff-Khnzorian (1980: 284, fig. 12.9).

### Subfamily Dinoderinae C.G. Thomson, 1863

#### Rhyzopertha dominica

Taxon classificationAnimaliaColeopteraBostrichidae

(Fabricius, 1792)

Rhyzopertha dominica : Hyde and Daubney 1960: 120.
Rhyzopertha dominica (Fabricius, 1792): Denux and Zagatti 2010: 348.

##### Literature records.

**Malta:** Floriana, St. Publius site, 1958, on grain, 1 ex (Hyde and Daubney 1960); “MT” [= Malta] (Denux and Zagatti 2010).

##### Material examined.

**Malta:** St. Thomas Bay, 27.VI.1990, DM, 1 ex (CMM); Zejtun, 20.VII.1989, DM, attracted to light, 1 ex (CMM); 15.IX.1989, DM, 1 ex (CNI); 21.IX.1989, DM, 1 ex (CMM); 28.IX.1989, DM, 1 ex (CMM); 10.IX.2001, DM, 1 ex (CMM); 24.XI.2001, DM, 2 ex (CMM; CNI); 3.VII.2002 DM, 1 ex (CMM).

##### Chorotype.

Cosmopolitan (Potter 1935, as *Rhizopertha dominica*, Borowski 2007). This species is widespread in the Palaeartic region (cf. Nardi 2004b, Borowski 2007). The followings territories were not included in its distribution provided by Borowski (2007): Bhutan (Taylor and Halliday 1986), Bulgaria (Zidan and Obretenchev 2009), China (Anhui, Chongqing, Guangdong, Guizhou, Hebei, Henan, Hubei, Hunan, Jiangxi, Shandong, Sichuan) (Lesne 1904, as *Rhizopertha dominica*, Song et al. 2012), Iran (cf. Abivari 2001, as *Rhizopertha dominica*, Ziaee et al. 2006, Ashouri and Shayesteh 2009), Israel (Halperin and Damoiseau 1980, as *Rhizopertha dominica*, Chikatunov et al. 2004, as *Rhyzoperta* [sic!] *dominica*, Chikatunov et al. 2006, as *Rhizopertha dominica*), Jordan (Sharaf et al. 1983), Libya (cf. Zanon 1922, Gridelli 1930, Zavattari 1934, in all cases as *Rhizopertha dominica*, Champ 1986), Lithuania (Ivinskis et al. 2009, as *Rhizopertha dominica*), Morocco (Martínez de la Escalera 1914, Rungs 1946, Español 1956b, Kocher 1956, in all cases as *Rhizopertha dominica*, Bartali et al. 1990, López-Colon 2000, as *Rhizopertha dominica*, Benhalima et al. 2004), Nepal (Taylor and Halliday 1986), Pakistan (cf. Quddus and Qayyum 1982, Ishaque et al. 1982, in both cases as *Rhizopertha dominica*, Taylor and Halliday 1986, Sardar Alam et al. 1999, Anwar et al. 2005, in both cases as *Rhizopertha dominica*, Wakil et al. 2006, Ahmed et al. 2008, as *Rhizopertha dominica*, Wakil et al. 2013), Portugal (Seabra 1943, as *Rhizopertha dominica*, Nardi 2004b, Bahillo de la Puebla et al. 2007, Baena and Zuzarte 2013), Romania (Ghizdavu and Deac 1995, Nardi 2004b), Saudi Arabia (Damoiseau 1979, Amin et al. 1986, in both cases as *Rhizopertha dominica*, Mostafa et al. 1981, Rostom 1993, Ahmed 1996), Switzerland (Hoppe 1981, Buchi 1993, Kenis 2005, as *Rhyzoperta* [sic!] *dominica*, Denux and Zagatti 2010), Turkey (Gerini 1971, Aydin and Soran 1987, Yucel 1988, Emekçi and Ferizli 2000), Ukraine (Podobivskiĭ 1991) and Uzbekistan (Asanov 1980).

##### Ecology.

Originally, this species was only associated with wood but is now considered as a primary pest of all kinds of stored grains. Both larvae and adults are able to attack whole grain, causing considerable damage. The species is thought to have originated from the Indian subcontinent, and was introduced worldwide by commerce. It is an economically important pest since it can cause signiﬁcant economic losses in terms of grain mass and nutrient depletion, and pose a public health risk from contamination by allergens, such as uric acid (cf. Potter 1935, Beeson and Bhatia 1937, Lepesme 1944, in all cases as *Rhyzopertha dominica*, Fisher 1950, Aitken 1975, Maes 1995, Pollini 1998, in both cases as *Rhyzopertha dominica*, Geis 2002, Gelosi and Süss 2001, Nguyen 2006, Borowski 2007, Liu et al. 2008, Denux and Zagatti 2010, Kenis and Branco 2010, Arthur et al. 2012, Edde 2012). This species can also damage books (Hoffman 1933, as *Rhyzopertha dominica*).

##### Notes.

The record from Malta by Hyde and Daubney (1960) was later overlooked by all authors (cf. Saliba 1963, Nardi 2004b, Borowski 2007). Thus this beetle must have been introduced in Malta at least since 1958 where it is now a well established species.

In Italy, *Rhyzopertha dominica* was first collected in Sicily during the nineteenth century (cf. Bertolini 1874, as *Rhyzopertha pusilla* Fabr. [= (Fabricius, 1798)]; Ragusa 1896, as *Rhyzopertha pusilla*), and since the 1950’s it was widespread in all regions (cf. Norato 1957, Dal Monte 1958, as *Rhyzopertha dominica*, Genduso 1963). According to Denux and Zagatti (2010: 348) the first European record of this species was from Czech Republic and is dated 1900, but the above Sicilian records, as well as those from other countries (cf. Reitter 1883, as *Rhyzopertha pusilla*, Lesne 1901b), are older. Moreover, this species has been present in Europe at least since 3500–2551 BP, as testified by its archelogical presence in Spain and Greece (cf. King 2009). So the species was introduced and established before 1492 A.D. at least in Spain and Greece and must be considered as parautocthonous (cf. Zapparoli 2008: 98). Illustrations of its male and female genitalia were provided by Potter (1935: 474–475, figs 21–25), Lesne (1945: 149, figs 9–13, as *Rhyzopertha dominica*) and Surtees (1961: 149, fig. 11, as *Rhyzopertha dominica*).

*Rhizopertha dominica granulipennis* Lesne in Beeson & Bhatia, 1937 from northern India (Uttarakhand, Chandi Randge) (Beeson and Bhatia 1937: 283, Lesne 1945: 146, as *Rhyzopertha dominica granulipennis*) was overlooked in recent catalogues (cf. Borowski 2007, Borowski and Węgrzynowicz 2007, Ivie 2010). In its original description – “A large form with exceptionally strongly developed granulation of the elytral declivity was bred from *Shorea robusta* [(Dipterocarpaceae)] and labelled *Rhyzopertha dominica granulipennis* by Lesne” (Beeson and Bhatia 1937: 283) – its rank is not unambiguously given, so, according to the Code (ICZN 1999, art. 45.6.4), it is an available subspecific name. Its authorship is here attributed to Lesne in Beeson and Bhatia (1937: 283), since Lesne (1945: 146) has reaffirmed the authorship of this name: “J’ai donné le nome de *Rhyzopertha dominica granulipennis* (1 [= footnote: Cf. Beeson et Bhatia, mémoire cité, p. 283.]) à cette forme remarquable” [= I named *Rhyzopertha dominica granulipennis* (1 [= footnote: Cf. Beeson et Bhatia, memory cited, p. 283.]) this remarkable form]. Unfortunately, it was not possible to study the type material of this taxon, that, according to Lesne (1945: 146), is housed in the Muséum National d’Histoire Naturelle of Paris (France) and in the Forest Research Institute of Derha Dun (India). However, on the basis of numerous specimens of this species from different territories (Nardi, unpublished data) which show a significant range of variability in the granulation of the elytral declivity, the following new synonymy, is here established: Rhizopertha dominica form granulipennis Lesne, in Beeson and Bhatia 1937: 283 = *Rhyzopertha dominica* (Fabricius, 1792: 359), **syn. n.**

### Subfamily Bostrichinae Latreille, 1802, Tribe Bostrichini Latreille, 1802

#### Amphicerus bimaculatus

Taxon classificationAnimaliaColeopteraBostrichidae

(A.G. Olivier, 1790)

##### Material examined.

**Malta:** Girgenti, 9.XII.2002, DM, 3 ♂♂ 4 ♀♀ (CMM; CNI); Zabbar, 29.IX.1995, DM, 1 ♀ (CMM); Zejtun, 29.V.1991, DM, 1 ♂ (CMM).

##### Other material examined.

[**Azerbaijan:**] Caucase, Elisabetpol [= Ganja], [no date], Babadjanides [leg.], 1 ♂ 1 ♀ (MCSVD).

##### Chorotype.

Turanic-Mediterranean except for Libya and Egypt, with extension into Portugal, Hungary, Tajikistan and Kyrgyzstan (cf. Borowski 2007, as Amphicerus (Caenophrada) bimaculata [sic!]). The occurrence of this species in Azerbaijan (Lesne 1899, 1905, Clermont 1909, Khalilov 1972, in all cases as *Schistoceros bimaculatus*, Ciampolini et al. 1989) is here confirmed. In fact, the distributional record of this species by Borowski (2007: 321), as “**E** [= Europe]: AF [= Afghanistan]”) is incorrect and should be “**E:** AB [=Azerbaijan]” (cf. Borowski and Węgrzynowicz 2007, as Amphicerus (Caenophrada) bimaculatus). A record from Iraq (Derwesh 1965, as *Schistoceros bimaculatus*) was later overlooked, while those from Germany (cf. Soro 1964, Zocchi 1971, in both cases as *Schistoceros bimaculatus*, Ciampolini et al. 1989, Pollini 1998) were never confirmed (cf. Lucht 1987, Köhler and Klausnitzer 1998, Geis 2002, Nardi 2004b, as *Schistoceros bimaculatus*, Borowski 2007, Borowski and Węgrzynowicz 2007). *Amphicerus bimaculatus* was intercepted at US ports, but it is not an established species in North America (Fisher 1950, as Amphicerus (Schistoceros) bimaculatus, Ivie 2002, Borowski and Węgrzynowicz 2007), while its establishment in Uruguay (cf. Soro 1964, Zocchi 1971, Pollini 1998), was not reported in recent publications (Borowski 2007, Borowski and Węgrzynowicz 2007, Barriga and Cepeda 2009).

##### Ecology.

Larval development of this species takes place mainly in dead wood of *Vitis* spp. and *Tamarix* spp., but other host plants are also recorded including: *Acacia* sp., *Annona cherimola*, *Cerasus* sp., *Citrus* sp., *Delonix regia*, *Ficus carica*, *Lycium* sp., *Malus communis*, *Olea europea*, *Prunus amygdalus*, *Punica granatum*, *Pyrus malus* and *Tamarindus indica* (cf. Lesne 1901b, as *Schistoceros bimaculatus*, Fisher 1950, Novak 1952, Caillol 1954, in both cases as *Schistoceros bimaculatus*, Español 1955, Kocher 1956, as *Schistoceros bimaculatus*, Soro 1964, Zocchi 1971, Halperin and Damoiseau 1980, Lundberg et al. 1987, in both cases as *Schistoceros bimaculatus*, Moleas 1988, Ciampolini et al. 1989, Ragusa and Russo 1989, fig. 8, as [sic!] *Apate monachus*, Pollini 1998, Akşit et al. 2005, Liberto and Audisio 2005, in both cases as *Schistoceros bimaculatus*, Bahillo de la Puebla et al. 2007, as Amphicerus (Caenophrada) bimaculata [sic!], Tezcan 2008, as *Schistoceros bimaculatus*).

**Notes.** First record for Malta. *Amphicerus bimaculatus* is not recorded from the neigbouring Sicilian islands (Tab. 1), but is known from mainland Sicily (cf. Audisio et al. 1995, Sparacio 1997, Nardi 2004b, in all cases as *Schistoceros bimaculatus*) and Tunisia (Borowski 2007). It is recorded also from two circumsardinian islands (Piras and Pisano 1972, as *Schistoceros bimaculatus*). Its aedeagus was figured by Iablokoff-Khnzorian (1976: 232, fig. 5, as *Schistoceros bimaculatus*).

**Table 1.** Bostrichidae recorded from Maltese Archipelago and neigbouring islands with respective chorotype codes. Abbreviations: A = Aitken 1975; AFM = Afrotropical-Mediterranean; C = Cameron and Caruana Gatto 1907; CEM = Centralasiatic-European-Mediterranean; COS = Cosmopolitan; D = Denux and Zagatti 2010; EUR = European; F = Falzon et al. 2012; G = Goggi 2004; H = Hyde and Daubney 1960; L = Luigioni 1929; LAMP. = Lampedusa; LINO. = Linosa; MED = Mediterranean; Na = Nardi 2004a; Nb = Nardi 2004b; NR = Nardi and Ratti 1995; PANTEL. = Pantelleria; SEU = S-European; TEM = Turanic-European-Mediterranean; TUM = Turanic-Mediterranean; WME = W-Mediterranean; ZGG = Chorotype; ! = this paper; () = misinterpretation.

Species	PANTEL.	LINO.	GOZO	MALTA	LAMP.	ZGG
*Lyctus brunneus*				C L Na D !		COS
*Trogoxylon impressum*	NR			!		TEM
*Rhyzopertha dominica*				H D !		COS
*Amphicerus bimaculatus*				!		TUM
*Bostrichus capucinus*	NR			A Nb		CEM
*Heterobostrychus aequalis*				!		COS
*Scobicia chevrieri*	NR		!	(C L Nb) F !	NR	MED
*Sinoxylon sexdentatum*	NR					MED
*Sinoxylon unidentatum*				!		COS
*Xylopertha praeusta*		G				WME
*Xyloperthella picea*				!		AFM
*Apate monachus*				!		AFM
Total/isLANDS	4	1	1	10	1	

The above specimens collected during 2002 are almost entirely black, probably for a *post mortem* colouration.

The correct grammatical gender for the specific name of this taxon is *bimaculatus*, since it was described as *Bostrichus bimaculatus* from “Provence” (southern France) (Olivier 1790: 109) and the above mentioned usage of *bimaculata* (Bahillo de la Puebla et al. 2007, Borowski 2007) is a subsequent incorrect spelling of this taxon (cf. Borowski 2013: 3).

The nomenclatorial problems for *Schistoceros* Lesne, 1899 were discussed by Ivie (2010).

#### Bostrichus capucinus

Taxon classificationAnimaliaColeopteraBostrichidae

(Linnaeus, 1758)

Bostrychus capucinus (L.): Aitken 1975: 8.
Bostrichus capucinus (Linnaeus, 1758): Nardi 2004b.

##### Literature records.

**Malta:** “Malta: wooden ornament” (Aitken 1975); “Malta” (Nardi 2004b).

##### Other material examined.

**Jordan:** Dana Reserve, El-Barrah, 1150 m, NE slope, 36R YU 517 926, 23.IV.–8.V.1995, D.P.I.T., Mediterranean environment, pt, 1 ex (MZUR); ditto, Wadi Araba Camp Site, 15–20.IV.1995, D.P.I.T., pt, 1 ex (MZUR). **Syria:** Palmyra, 15.VII.2003, G. Serra leg., 1 ex (MZUF).

**Chorotype.** Centralasiatic-Mediterranean, including parts of Northern Europe (cf. Nardi and Ratti 1995, as *Bostrychus capucinus*, Borowski 2007), the Algerian Sahara (Lesne 1899, 1901b, both as *Bostrychus capucinus*) and coastal Sudan (Cloudsley-Thompson 1962, as *Bostrychus capucinus*). This species reaches Altai mountains (Lesne 1901b, Borowski and Węgrzynowicz 2007), Asian Kazakhstan (Borowski 2007), Kyrgyzstan (Vrydagh 1956, Ovtchinnikov 1996, in both cases as *Bostrychus capucinus*), Tajikistan (Lesne 1901b), Northwest China (Lesne 1904, Horion 1961, in both cases as *Bostrychus capucinus*, Borowski 2007), China (without further details) (Borowski 2007, Yan et al. 2010, as *Bostrychus capucinus* and Bostrychus capucinus var. rubrirenttis [sic!] Zouf [= *rubriventris* Zoufal, 1894]) and eastern Siberia (Borowski 2007). It was intercepted numerous times at US ports, but is not yet established in North America (cf. Fisher 1950, Ivie 2002). The above record from Jordan is the first for this country (cf. Sharaf et al. 1983, Borowski 2007), even though the presence of this species in Jordan was expected because of its occurence in neibouring countries (e.g. Israel, Syria, etc.); in Israel the species was probably introduced with timber from Europe (Bytinsky-Salz 1966, Bytinski-Salz and Sternlicht 1967, Halperin and Damoiseau 1980, in all cases as *Bostrychus capucinus*, Borowski 2007).

##### Ecology.

This species develops chiefly in the wood of oaks (*Quercus ilex*, *Quercus robur*, *Quercus toza*, etc.), but is also recorded from many other broadleaves trees and scrubs such as *Pinus*, and timber (cf. Nardi and Ratti 1995, Sparacio 1997, Liberto and Audisio 2005, Bahillo de La Puebla et al. 2007, Baena and Zuzarte 2013). Occasionally it can produce house infestations (Saccà 1940, as *Bostrychus capucinus* Geoffroy [sic!], Cavalloro and Ratti 1978, Lodos 1985, Hellrigl 2006, in all cases as *Bostrychus capucinus*).

##### Notes.

The above record of Aitken (1975) was based on material collected in UK from cargo originating from Malta. The presence of this species in Malta needs to be confirmed. Although *Quercus ilex* is present in Malta, its abundance is very scarce on the archipelago (cf. Haslam et al. 1977, Schembri 1993, 1997). *Bostrichus capucinus* is known from other similarly small Mediterranean islands, such as the nearby Pantelleria Island, three circumsardinian islands (Piras and Pisano 1972, as *Bostrychus capucinus*, Nardi and Ratti 1995), two northern Adriatic islands (Müller 1923: 28, Schatzmayr and Müller 1925: 74, Luigioni 1929: 611, in all cases as *Bostrychus capucinus*), and the Balearic Islands (cf. Español 1955, as *Bostrychus capucinus*).

#### Heterobostrychus aequalis

Taxon classificationAnimaliaColeopteraBostrichidae

(Waterhouse, 1884)

##### Material examined.

**Malta:** Rabat, 21.IX.2001, PS, 1 ♂ (CMM).

##### Chorotype.

A cosmopolitan species of Indo-Malaysian origins. It is mainly distributed in tropical and sub-tropical regions and restricted to 40° north and south of the equator (cf. Borowski 2007, Borowski and Węgrzynowicz 2007, Azmi et al. 2011: 500, fig. 2). In the Mediterranean, it was intercepted in Israel, Italy and Spain (cf. Gambetta 1983, Geis 2002, Ratti 2002, 2004b, Bahillo de la Puebla et al. 2007, Ratti 2007, Azmi et al. 2011). Ireland (O’Mahony 1949, as *Heterobostrichus* [sic!] *aequalis*), France (cf. Brustel and Aberlenc 2014) and Oregon (Westcott et al. 2006) must be added to the countries in which this species was intercepted (cf. Azmi et al. 2011), eventhough the former record was based on collection of death specimens (larvae and adults), which was later ignored (cf. Anderson et al. 1997, Geis 2002, Nardi 2004b, Borowski 2007, Denux and Zagatti 2010, Alexander and Anderson 2012).

##### Ecology.

Polyphagous species attacking some 36 unrelated host-plant genera; this species breeds not only in logs, but also in planks, furniture, plywood and roots of manioc (cf. Fisher 1950, Kalshoven 1963a, 1963b, Horion 1972, Gambetta 1983, Wang et al. 1996, as *Heterobostrachus* [sic!] *aequalis*, Geis 2002, Maes 2005, Aguilera 2006, Bahillo de la Puebla et al. 2007, Sitticaya et al. 2009, Robinson 2013).

**Notes.** First record for Malta. This species become established in some countries where it was accidentally introduced. Temperatures of 17 °C and below are said to be unsuitable for the species to breed (cf. Ivie 2002, Azmi et al. 2011). Thus, considering the warm climate of the Maltese Islands, (Chetcuti et al. 1992), it is highly likely that the species is already an established one. The above specimen was collected with a light trap on a terrace, mainly surrounded by agricultural land (P. Sammut, pers. comm., 2002).

### Subfamily Bostrichinae Latreille, 1802, Tribe Sinoxylini Marseul, 1857

#### Sinoxylon unidentatum

Taxon classificationAnimaliaColeopteraBostrichidae

(Fabricius, 1801)

##### Material examined.

**Malta: Malta:** Marsa, Għammieri, 24.I.2007, DM, taken from wood packaging material originating from India, 1 ex (CMM).

##### Chorotype.

A cosmopolitan species native to tropical eastern Asia, and widespread in the intertropical regions of the world. In Europe, it was intercepted in France, Germany, Great Britain, Italy, Poland, Russia, Spain and Ukraine, but its establishment was never confirmed. Moreover, the species is not reported from North Africa and the Middle East (except from Yemen) (cf. Vrydagh 1955, Poggi et al. 1994, Peck et al. 1998, Geis 2002, Ratti 2004, Maes 2005, Iwata and Nakano 2006, Liu et al. 2006, Peres Filho et al. 2006, Westcott et al. 2006, in all cases as *Sinoxylon conigerum* Gerstäcker 1855, Bahillo de la Puebla et al. 2007, Borowski 2007, Borowski and Węgrzynowicz 2007, Barriga and Cepeda 2009, as *Sinoxylon conigerum*, Savoldelli and Regalin 2009, Price et al. 2011, Brustel and Aberlenc 2014).

##### Ecology.

In its place of origin, *Sinoxylon unidentatum* develops in the wood of many unrelated plant families mainly: Anacardiaceae, Combretaceae, Dipterocarpaceae, Euphorbiaceae, Lamiaceae, Lauraceae, Leguminosae, Mimosaceae, Myrtaceae, Rubiaceae, Tiliaceae, Ulmaceae, etc. (cf. Poggi et al. 1994, Peres Filho et al. 2006, as *Sinoxylon conigerum*, Bahillo de la Puebla et al. 2007, Savoldelli and Regalin 2009).

##### Notes.

This alien species is a new record for the central Mediterranean area. Due to its polyphagy, it is frequently exported with wooden packing material (cf. Bahillo de la Puebla et al. 2007). The Maltese climate (Chetcuti et al. 1992, Schembri 1997) is unsuitable for its establishment (J. Borowski, pers. comm. 2012), but in an indoor site of northern Italy, this species was able to complete its development and to spread the infestation (Savoldelli and Regalin 2009).

### Subfamily Bostrichinae Latreille, 1802, Tribe Xyloperthini Lesne, 1921

#### Scobicia chevrieri

Taxon classificationAnimaliaColeopteraBostrichidae

(A. Villa & J.B. Villa, 1835)

Xyloperta pustulata F.: Cameron and Caruana Gatto 1907: 398.
Scobicia pustulata Fabr.: Luigioni 1929: 641.
Scobicia pustulata (Fabricius, 1801): Nardi 2004b.
Scobicia chevrieri (A. Villa & J.B. Villa, 1835): Mifsud et al. 2012: 9.

##### Literature records.

**Malta:** [Malta,] “Coll. Gatto” (Cameron and Caruana Gatto 1907); “Mal.” [= Malta] (Luigioni 1929); “Malta” (Nardi 2004b); Buskett, adults emerged between 5–25.X.2011 from dead twigs of *Ficus carica* collected on 8.VII.2011, AF & DM leg., 56 ex (Mifsud et al. 2012).

##### Material examined.

**Gozo:** Marsalforn Valley, 6.VI.1990, DM, 1 ex (CMM). **Malta:** Baħrija, 5.VIII.1992, LC, 1 ex (CMM); Bingemma, 10.IX.2001, DM, attracted to light, 8 ex (CMM); Buskett, 24.VI.2003, DM, attracted to light, mixed woodland *Pinus*/*Cupressus*, 5 ex (CMM); Marsa, Għammieri, 24.III.2002, DM, 1 ex (CMM); “Malta, 9/[= IX.]1901”, “Xylopertha”, “pustulata”, “M. Cameron Coll. / B.M. 1936-555”, “5777” [= *Xylopertha pustulata* F. Marsa Scirocco [= Marsaxlokk]/id. EAN [= ?; maybe identified by E. A. Newbery (cf. Cameron and Caruana Gatto 1907: 383)]], 10 ex (BMNH); Rabat, 14.VI.2002, PS, 1 ex (CMM); 3.VIII.2002, PS, 3 ex (CMM); Rabat, Dwejra, 21.VI.2002, PS, 1 ex (CMM); Rabat, Ta Koronja, 6.VI.2002, PS, 2 ex (CMM); Wied Badu, 3.VII.2002, DM, 5 ex (CMM); Wied tal-Isqof, 16.VII.2002, DM, 1 ex (CMM); 2.VIII.2002, DM, 2 ex (CMM); Zejtun, 10.XI.1989, DM, 1 ex (CMM); 29.IX.1990, DM, development of larvae took place in dead branches of vines, 2 ex (CMM); 27.V.2002, DM, 1 ex (CMM).

##### Other material examined.

**Italy:** Marche region, Ancona prov., Gole di Frasassi, 20.VI.2001, A. B. Biscaccianti leg., ex [larvae from] *Corylus avellana*, 2 ex (CNI); ditto, Pesaro e Urbino prov., Foce Fiume Metauro, area golenale [= Mouth of Metauro River, floodplain area], 2.VI.1999, A. B. Biscaccianti leg., ex larvae from *Salix* sp., 4 ex (CNBFVR; CNI). Latium region, Rome prov., Tenuta Presidenziale di Castelporziano, Ponte della Focetta, 10.IX.1997, A. B. Di Giulio leg., hygrophilous wood, light trap 15 W, 1 ex (CNI); ditto, ditto, ditto, Villa di Capocotta, 21.VI.2000, P. Maltzeff leg., mixed light trap 160 W, 2 ex (CNI); ditto, Latina prov., Cisterna di Latina, [33T 319824.15 E 4606546.61 N], 29.IX.1987, GN, night, in a garden, at light, 2 ex (CNI); ditto, ditto, ditto, Fraz. Cerciabella, [33T 319479.96 E 4605030.39 N], 17.VIII.1998, GN, in a garden, at light, 20–21 hours, 2 ex (CNI). **Jordan:** Dana Reserve, Acacia Area, 17.IV.1995, D.P.I.T., night catch, 1 ex (MZUR); ditto, El-Barrah, 1150 m, NE slope, 36R YU 517 926, 23.IV.–8.V.1995, D.P.I.T., Mediterranean environment, pt, 1 ex (MZUR); ditto, Irano Turanian Area 1, 18.IV.1995, D.P.I.T., 1 ex (MZUR).

##### Chorotype.

Mediterranean (northward upto Austria, French Alps, Hungary and Switzerland), with extension westward upto Portugal, and eastward upto Azerbaijan, Georgia, Iran and southern Russia; this species was intercepted in the USA and Canada, but so far it has not established itself (cf. Fisher 1950, as *Sinoxylon chevrieri* (Villa) [sic!], Vrydagh 1952, as *Sinoxylon Chevrieri* (Villa) [sic!], Ivie 2002, Borowski 2007, Borowski and Węgrzynowicz 2007, McCaffrey 2011, as *Sinoxylon chevrieri* Villa & Villa, 1835 [sic!]). It is known also from Romania (Lesne 1904, Vrydagh 1956, in both cases as *Sinoxylon Chevrieri* Villa [sic!], Nardi 2004b) and Sinai (cf. Alfieri 1976, as *Sinoxylon chevrieri* Villa [sic!]), but these two regions were overlooked by Borowski (2007). The above record from Jordan is the first for this country (cf. Sharaf et al. 1983, Borowski 2007).

##### Ecology.

Polyphagous species, with development taking place in death or debilitated branches of several woody plants. The following are plants known to be infested by this species and are present in Malta (cf. Haslam et al. 1977): *Acacia* sp., *Amygdalus communis*, *Arundo* sp., *Bambusa* sp., *Ceratonia siliqua*, *Cercis* sp., *Citrus* sp., *Eucalyptus* sp., *Ficus carica*, *Hibiscus sabdariffa*, *Laurus nobilis*, *Morus alba*, *Olea* sp., *Pinus halepensis*, *Pistacia lentiscus*, *Pistacia vera*, *Prunus avium*, *Pistacia dulcis*, *Punica granatum*, *Quercus* spp., *Rhamnus alaternus*, *Ulmus* sp. and *Vitis* spp. (cf. Lesne 1901b, as *Scobicia Chevrieri* Villa [sic!], Peyerimhoff 1919, Novak 1952, both as *Scobicia Chevrieri* (Villa) [sic!], Caillol 1954, Español 1955, as *Scobicia chevrieri* Vill. [sic!], Bytinski-Salz and Sternlicht 1967, as *Scobicia chevrieri* Villa [sic!], Compte 1970, as *Scobicia chevrieri* (Villa [sic!], 1835), Georghiou 1977, as *Scobicia chevrieri* Villa [sic!], Halperin and Damoiseau 1980, as *Scobicia chevrieri* (Villa) [sic!], Lundberg et al. 1987, as *Scobicia chevrieri* Villa [sic!], Nardi and Ratti 1995, Borowski and Mazur 2001, Nardi and Zahradník 2004, Akşit et al. 2005, as *Scobicia chevrieri* Villa [sic!], Baena and Zuzarte 2013).

In central Italy (Marche region), this species (see above) was reared also from wood of *Salix* sp. and *Corylus avellana*, that represent new host-plant records for this Bostrichid (see above listed literature).

This species is often collected at light (Angelini 1996a, 1998, Chikatunov et al. 2006, Baena and Zuzarte 2013) and by window flight traps. Using these traps, large number of specimens were collected in forests of *Quercus calliprinos*, *Pinus halepensis* and *Pinus brutia* from northern Israel (Buse et al. 2010), in *Quercus suber* forests from southern France (Brin et al. 2005, Brin and Brustel 2006), in an oak-hornbeam forest (*Querco*-*Carpineto boreoitalicum*) from northern Italy (Nardi and Zahradník 2004), in *Quercus ilex* forests of Sardinia, in a floodplain remnant of northern Italy (Nardi, unpublished data) and in mixed beechwoods of central Italy (Redolfi De Zan et al. 2014).

This species was also recorded from urban areas (Nardi 1997, Inglebert 2004).

##### Notes.

*Scobicia pustulata* (Fabricius, 1801), a closely related Mediterranean species (cf. Borowski 2007), is here excluded from the Maltese fauna, since the record by Cameron and Caruana Gatto (1907) should refer to *Scobicia chevrieri* as established by the examination of the above mentioned historical material. This is not a case of misidentification by Cameron and Caruana Gatto (1907), since they (Cameron and Caruana Gatto 1907: 383) based the nomenclature of this species on Heyden et al. (1891: 467) who listed “*Xyloperta pustulata* F.Kiesw.”, and “*Xyloperta pustulata* Kiesenw. (non F.)” is *Scobicia chevrieri* (Lesne 1938: 57, as *Scobicia Chevrieri* Villa [sic!], 1835). Only on the basis of this old litterature record, *Scobicia pustulata* was erroneously listed from Malta by the above subsequent authors (Luigioni 1929, Nardi 2004b).

*Scobicia chevrieri* is a good colonizer of Mediterranean islands, since it is recorded also from other islands such as Montecristo (Tuscan Archipelago), Pantelleria, Lampedusa (cf. Nardi and Ratti 1995), Balearic and Columbretes Islands (cf. Lesne 1901b, as *Scobicia Chevrieri* Villa [sic!], 1835, Español 1955, Vrydagh 1960b, as *Scobicia chevrieri* Villa [sic!], 1835, Compte 1970, Nardi 2004b).

#### Xyloperthella picea

Taxon classificationAnimaliaColeopteraBostrichidae

(A.G. Olivier, 1790)

##### Material examined.

**Malta:** Qormi, Ħal-Farrug, 5.V.2003, DM [under bark of *Ceratonia siliqua* in an agricultural environment], 1 ♀ (CMM); Rabat, 4.VI.1989, PS, attracted to light [on the roof of private residence], 1 ex (CMM); 18.VI.1992, PS, [attracted to light, in an agricultural environment], 2 ex (CMM); 4.VI.1999, PS, attracted to light [in an agricultural environment], 1 ♂, (CMM); 28.VI.2001, PS, [attracted to light, in an agricultural environment], 1 ♀ (CMM); 21.VI.2002, PS, [ditto], 2 ♂♂ (CNI); 23.VI.2002, PS, [ditto], 2 ♀♀ (CMM; CNI); 1.VII.2002, PS, [ditto], 1 ♂ 1 ♀ (CMM); 3.VI.2003, PS, [ditto] 1 ♀ (CNI); Rabat, Ta Koronja, 21.VI.2002, PS, [attracted to light], 1 ♀ (CMM).

##### Other material examined.

**Italy:** Apulia region, Lecce prov., S. Cataldo, Ris. [= Riserva = Reserve] WWF Le Cesine, 11–21.VI.1995, F. Angelini, 2 ex (MCSVA). Sardinia region, Sassari prov., Berehidda, 15.VII.1985, M. Daccordi leg., 1 ex (MCSV). Sicily region, Siracusa prov., Noto, Oasi di Vendicari, Cala Mosche, 10 m, N°36 49,066’ E15°5,834’, 3.VII.2011, D. Birtele & P. Birtele leg., 1 ♀ (CCI).

##### Chorotype.

Afrotropical-Mediterranean species which was also intercepted in Germany, Great Britain and The Netherlands (cf. Lesne 1924, 1938, in both cases as *Xylopertha picea*, Aitken 1975, Akşit et al. 2005, Borowski 2007, in both cases as *Xyloperthella picea picea*, Bahillo de la Puebla et al. 2007, Borowski and Węgrzynowicz 2007, Baena and Zuzarte 2013). According to several authors (cf. Lesne 1901a, 1924, 1938, Gridelli 1939, 1940, Blackwelder 1945, Da Costa Lima 1953, in all cases as *Xylopertha picea*, Vrydagh 1958, 1960a, 1960b, 1961, 1962, Geis 2002, Ivie 2002, Borowski 2007, Peres Filho et al. 2007, Barriga and Cepeda 2009, Baena and Zuzarte 2013), it became established in parts of the Neotropical region (Argentina, Brazil, Colombia, French Guyana, Jamaica, Paraguay, Perù) since a long time but its presence in this region was recently reported as doubtful (Borowski and Węgrzynowicz 2007).

##### Notes.

First record for Malta and the Apulia region; the species is not known from neighbouring Sicilian Islands (Tab. 1) but is widespread in Africa. Whether this species is autochthonous or has been introduced into Malta might be therefore open to debate. It is known from Tunisia and from all other mainland countries of North Africa (Lesne 1901b, Normand 1936, in both cases as *Xylopertha picea*, Vrydagh 1956, Borowski 2007), while from southern Europe it is recorded only from Portugal (cf. Serrano 1981, as *Xylopertha picea*, Baena and Zuzarte 2013), Greece (Samos Island) (Vrydagh 1962, Nardi 2004b, as *Xyloperthella picea picea*), southern mainland Italy (Basilicata region), Sardinia, Sicily, Spain and the Balearic Islands (cf. Lesne 1901a, 1901b, 1905, Winkler 1927, Luigioni 1929, Porta 1929, in all cases as *Xylopertha picea*, Cobos 1950, as *Xyloperthe* [sic!] *picea*, Español 1955, 1974, Angelini and Montemurro 1986, Audisio et al. 1995, Sparacio 1997, Angelini 1998, Nardi 2004b, Bahillo de la Puebla et al. 2007, Borowski 2007).

According to some authors (cf. Lesne 1924, Español 1955), the Mediterranean populations may belong to a distinct subspecies, *Xyloperthella picea heydeni* (Schilsky, 1899), nevertheless the species is currently monotypic since this subspecies and also *Xyloperthella picea plumbeipennis* Lesne, 1924 from Gabon and Democratic Republic of Congo (Lesne 1924), were recently listed as a synonym of *Xyloperthella picea* (Borowski and Węgrzynowicz 2007). The species was described from Cape Verde Islands (Olivier 1790, as *Bostrichus piceus*) and not from Senegal as indicated by some authors (López-Colón 2000, López-Colón et al. 2001, Bahillo de la Puebla et al. 2007); in this African archipelago it is probably autochthonous (Oromí et al. 2005).

### Subfamily Apatinae Billberg, 1820, Tribe Apatini Billberg, 1820

#### Apate monachus

Taxon classificationAnimaliaColeopteraBostrichidae

Fabricius, 1775

##### Material examined.

**Malta:** Għargħur, 18.VII.2013, DM, on living branchs of *Ficus carica*,1 ♂ (CMM); Manikata, VII.2012, DM, in healthy branch (3–5 cm in diameter) of *Ceratonia siliqua* tunneled by this beetle, 1 ♀ death (CMM). Mellieħa, Kortin, 24.VII.2004, HB, U.V. light trap, 1 ♀ (CNI); 28.VII.2004, HB, U.V. light trap, 2 ♀♀ (CMM); 29.VII.2004, HB, U.V. light trap, 1 ♂ (CMM); 15.VIII.2004, HB, U.V. light trap, 1 ♂ (CMM); 28.VI.2005, HB, U.V. light trap, 1 ♀ (CMM); 3.VII.2005, HB, U.V. light trap, 1 ♂ (CNI); 5.VII.2005, HB, UV lights, 1 ♀ (CNI); 16.VII.2005, HB, U.V. light trap, 2 ♀♀ (CMM; CNI); 18.VI.2006, HB, UV lights, 1 ♂ 1 ♀ (CMM); 20.VI.2006, HB, UV lights, 1 ♀ (CNI); 2.VII.2006, HB, UV lights, 1 ♀ (CMM); 19.VI.2009, HB, UV lights, 1 ♂ (CMM); 5.VII.2009, HB, UV lights, 3 ♂♂ 1 ♀ (CMM; CNI); 11.VIII.2009, HB, UV lights, 2 ♂♂ (CMM); 13.VIII.2009, HB, UV lights, 1 ♂ (CNI); 22.VIII.2009, HB, UV lights, 1 ♂ (CMM); Mellieħa, Santa Maria Estate, 24.VII.2004, HB, 1 ♀ (CMM).

##### Other material examined.

**Italy:** Calabria region, [Cosenza prov.,] Sibari, VII.1924, G. Leoni leg., 1 ex (MCZRL) (cf. Luigioni 1929: 642). Sardinia region, Cagliari prov., Geremeas, 18.VIII.2001, LF, 1 ♀ (CNI); ditto, ditto, [Island of Sant’Antioco,] Sant’Antioco, Torre Canai, 25.VIII.[19]79, Ferrara leg., 1 ♂ (MZUR); ditto, Oristano prov., Arborea, S. Anna, 10.VI.2004, LF, 6 ♂♂ 1 ♀ (CNI); ditto, Nuoro prov., Orosei, VI.1956, E. De Maggi leg., 1 ex (MCZRD); ditto, [Nuoro prov., Siniscola,] Capo Comino, 31.VIII.1973, L.G. Donadini leg., 1 ♀ (MCSV); ditto, [Sassari prov.,] Stintino, Punta Negra, 15.VII.1998, G. Mambrini leg., 1 ♂ (CCI). **France:** Corsica, Bastia, Pineto, 3.VIII.[19]80, A. Sette leg., 1 ♀ (MCSV).

##### Chorotype.

Afrotropical-Mediterranean (northward upto Corsica and northern Spain). This species is established in the Neotropical region (Greater Antilles, Brazil, etc.), and was intercepted in southern France, in central European countries and USA (cf. Vrydagh 1960b, Reichardt 1964, Horion 1972, Aitken 1975, Geis 2002, fig. 31, Ivie 2002, Nardi 2004b, Bahillo de la Puebla et al. 2007, Borowski 2007, Barriga and Cepeda 2009, Ciesla 2011, Brustel and Aberlenc 2014).

##### Ecology.

The genus *Apate* Fabricius, 1775 is one of the most notorious and troublesome forest pests in Africa (cf. Schabel 2006). *Apate monachus* is a polyphagous beetle, with over 80 host-plants used for larval development (cf. Lesne 1924, Rungs 1946, Boselli 1959, as Apate monachus var. rufiventris P.H. Lucas, 1843, Peretz and Cohen 1961, Boselli 1962, as *Apathe* [sic!] *monachus*, Chararas and Balakowski 1962, Prota 1963, Zanardi et al. 1969, Zocchi 1971, Halperin and Damoiseau 1980, Luciano 1982, Benfatto and Longo 1985, Sadok and Gerini 1988, Borowski and Mazur 2001, Gobbi 2003, Pisano et al. 2003, Schabel 2006, Bahillo de la Puebla at al. 2007, Di Franco and Benfatto 2008, Bonsignore et al. 2011, Ciesla 2011, Bonsignore 2012, Cillo and Bazzato 2012) of which, the following occur also in Malta (E. Lanfranco, pers. comm., 2014): *Acacia* spp., *Ailanthus glandulosa*, *Amigdalis comunis*, *Arbutus unedo*, *Armeniaca vulgaris*, *Ceratonia siliqua*, *Citrus bigaradia*, *Citrus limon*, *Citrus limonia*, *Citrus nobilis*, *Citrus sinensis*, *Cupressus* sp., *Erica* sp., *Erythrina* sp., *Eucalyptus* spp., *Grevillea* sp., *Malus communis*, *Malus domestica*, *Melia azedarach*, *Myrtus comunis*, *Olea europaea*, Olea europaea var. oleaster, *Persica vulgaris*, *Phoenix dactylifera*, *Pinus pinea*, *Pisidium guajava*, *Pistacia lentiscus*, *Pyrus amigdaliformis*, *Pyrus dulcis*, *Pyrus communis*, Pyrus communis var. piraster, *Prunus amygdalus*, *Pyrus armeniaca*, *Pyrus domestica*, *Pyrus persica*, *Pyrus spinosa*, *Punica granatum*, *Quercus ilex*, *Schinus* sp., *Tamarix* sp., and *Vitis* spp. Adults are nocturnal and as observed also in Malta, they are frequently collected at light (cf. Lesne 1924, Sparacio 1997, Angelini 1998, Ragusa and Russo 1989, Chikatunov et al. 2006, Bahillo de la Puebla et al. 2007). In the Afrotropical region, *Lyctoderma africanum* (Grouvelle, 1900) and *Lyctoderma testaceum* Lesne, 1913 (Bostrichidae, Lyctinae) are associated with adults of *Apate monachus*, usually living under the abdomen and between the legs of these beetles (Lesne 1932, Paulian 1988: 501).

##### Notes.

First record for Malta and it is not known from neighbouring Sicilian Islands (Tab. 1). Most of the Maltese specimens were collected at Mellieħa, Kortin using U.V. lights, with sex ratio of 1:1. This habitat can be best described as garigue but with pockets of low lying *Ceratonia siliqua* and *Pistacia lentiscus*. At Manikata, several healthy branches (3–5 cm in diameter) of *Ceratonia siliqua* were found tunneled by this beetle; the wood was drilled in the late summer, and the above mentioned specimen was found death in one of these holes. This area was recently converted into an agritouristic area and the owners were very concerned when they found the healthy branches of *Ceratonia siliqua* damaged by this beetle. According to the above records, the earliest capturs of this species from Malta was 2004. This beetle is very conspicuous (10–19 mm of lenght: Bahillo de la Puebla et al. 2007) and it may be hypothesised that it is a recently established species in Malta. Having said this however, it is also worth mentioning that very few people in Malta were interested in collecting and studying these beetles in recent years. *Apate monachus* is also recorded from Tunisia (Lesne 1924, Borowski 2007) and Italy. In Italy it is known from three southern mainland regions (Apulia, Basilicata and Calabria), Sicily and Sardinia (including some small circumsardinian islands) (cf. Dodero 1908, as Apate monachus ab. rufiventris, Luigioni 1929, Porta 1929, as Apate monachus a. rufiventris, Boselli 1959, 1962, Chararas and Balachowsky 1962, Prota 1963, Tassi 1967, Zanardi et al. 1969, Zocchi 1971, Luciano 1982, Benfatto and Longo 1985, Ragusa and Russo 1989, Audisio et al. 1995, Angelini 1996b, Sparacio 1997, Angelini 1998, Gobbi 2003, Pisano et al. 2003, Bonsignore et al. 2011, Bonsignore 2012, Cillo and Bazzato 2012). Its doubtfull presence “N?”, in northern Italy (Audisio et al. 1995) was later confirmed by records from South Tyrol (Kahlen and Hellrigl 1996) that are probably based only on interceptions, since its establisment in the mentioned Alpine region is climatically improbable (Nardi, unpublished data).

The aedeagus of this species was figured by Jeannel and Paulian (1944: 91, figs 75, 87) and by Jeannel (1955: 57, fig. 32c).

The nomenclatorial problems for *Apate* Fabricius, 1775 were discussed by Borowski and Węgrzynowicz (2009).

## Discussion

Table 1 summarized the species of Bostrichidae recorded from the Maltese Archipelago and neigbouring Sicilian Islands with each assigned to a chorotype. Altogether 12 species are known: 4 from Pantelleria, 1 from Linosa, 1 from Gozo, 9 from Malta and 1 from Lampedusa; and only *Scobica chevrieri* is recorded from four islands (except from Linosa).

For Sicily and Tunisia, 22 (Catara and Barbagallo 1980, Benfatto and Longo 1985, Audisio et al. 1995, Nardi 2004b, 2004b, Suma and Russo 2005, Muscarella et al. 2013) and 30 species respectively (Borowski 2007) of Bostrichidae are recorded. The relatively small number of species recorded from the Sicilian Channel Islands (Tab. 1) is probably due to the limited surface area, by the absence of many host plants, and the absence of certain habitats such as moutain and sub-mountain ranges.

Excluding *Sinoxylon sexdentatum* (A.G. Olivier, 1790) (= *muricatum* (Linnaeus, 1767) *nomen oblitum*) and *Xylopertha praeusta* (Germar, 1817), Malta hosts all species recorded from the Sicilian Channel Islands (Tab. 1). Some of the host plants (e.g. *Vitis* spp., *Pistacia* spp., *Ficus carica*, *Malus domestica*, *Olea europaea*, *Prunus persica*, *Pyrus communis*, *Quercus ilex*, *Ceratonia siliqua*, *Cercis siliquastrum*, *Citrus* spp.) of the former species (cf. Lesne 1901b, 1906, Peyerimhoff 1919, Halperin and Damoiseau 1980, Gobbi 1984, Benfatti and Longo 1985, Moleas 1988, Mart et al. 1995, Ratti and Nardi 1995, Mourikis et al. 1998, Pollini 1998, Akşit et al. 2005) are present also in Malta (cf. Borg 1962, Saliba 1963, Haslam et al. 1977), so this polyphagous beetle could be eventually found in Malta or may establish itself. The occurrence of *Xylopertha praeusta*, which develops chiefly in the wood of oak trees (*Quercus ilex*, *Quercus suber*, *Quercus robur*, *Quercus mirbecki*) (Lesne 1901b, as *Xylonites praeustus* Germar, Rungs 1946, as *Xylopertha praeustus* Germ., Español 1955, Bahillo de la Puebla et al. 2007), is less probable since oak trees are rare in the Maltese Islands.

With the current available data, no conclusions can be drawn on how the bostrichid fauna colonised the Maltese Islands, but we can say that almost all species are also known from Sicily and North Africa, and are normally widely spread in the Mediterranean area. In fact, from a zoogeographical point of view (cf. Tab. 2) wide chorotypes prevails: 40% Cosmopolitan, 30% Palaearctic, 20% Afrotropical-Mediterranean and 10% Mediterranean. European chorotypes are absent; this situation was observed in other families of Coleoptera (Mifsud and Bílý 2002, Nardi and Mifsud 2003, Háva and Mifsud 2006), whereas they are rappresented in two other families of saproxylic beetles (Mifsud 2002, Mifsud and Knížek 2009).

**Table 2.** Chorotypes of the Bostrichidae from Malta Archipelago and neigbouring islands, their distribution and percents (abbreviations as in Table 1).

Chorotypes	Number of species (%)	Pantelleria	Linosa	Maltese Islands	Lampedusa
COS	4 (33.33)			4 (40)	
AFM	2 (16.66)			2 (20)	
CEM	1 (8.33)	1 (25)		1 (10)	
TEM	1 (8.33)	1 (25)		1 (10)	
TUM	1 (8.33)			1 (10)	
MED	2 (16.66)	2 (50)		1 (10)	1 (100)
WME	1 (8.33)		1 (100)		
Total	12	4	1	10	1

Species of bostrichids are easly transported with infested woods and cereal, and are frequently observed in harbour areas (cf. Aitken 1975, Contessi 1991, Contessi and Mucciolini 1993, 1998, Peck et al. 1998, Haack and Cavey 2000, Geis 2002, Nicoli Aldini 2003, 2004, Ratti 2004, Borges et al. 2006, Haack 2006, Liu et al. 2006, Majka 2007, Ratti 2007, Beiriger 2010, Humble 2010, Leal et al. 2010, Price et al. 2011). The interception in Malta of other alien species from warm climatic regions is highly likely, and their possible establishment could also be promoted by global warming (cf. Parmesan 2006, Franceschini et al. 2009, Walther et al. 2009, Fronzek et al. 2010, Robinet and Roques 2010, Roques 2010, Thuiller et al. 2011).

From a faunistic point of view, six species are here recorded for the first time, namely: *Trogoxylon impressum*, *Amphicerus bimaculatus*, *Heterobostrychus aequalis*, *Sinoxylon unidentatum*, *Xyloperthella picea* and *Apate monachus*.

The Mediterranean area is one of the most signiﬁcantly altered biodiversity hotspots on Earth, since it has been intensively affected by human activity for millennia. As a result, only 4.7% of its primary vegetation remained unaltered and the landscape has been repeatedly transformed (cf. Myers et al. 2000, Cuttelod et al. 2008, Geri et al. 2010, Carroll et al. 2012). This transformation has negatively influenced xylophagous insect populations, especially in small and isolated environments, like those of islands (cf. Becker 1975, Howart and Ramsay 1991, Becker 1992, Crisp et al. 1998, Mantisi 2001, Pasta and La Mantia 2002). In this framework, half of bostrichid species recorded from the Maltese Islands (*Amphicerus bimaculatus*, *Bostrichus capucinus*, *Scobicia chevrieri*, *Xyloperthella picea*, *Apate monachus*), are included in the European Red list of saproxylic beetles (Nieto and Alexander 2010: 29), but luckily all belong only to the IUCN category “LC” (Least Concern).

## Supplementary Material

XML Treatment for Lyctus brunneus

XML Treatment for Trogoxylon impressum

XML Treatment for Rhyzopertha dominica

XML Treatment for Amphicerus bimaculatus

XML Treatment for Bostrichus capucinus

XML Treatment for Heterobostrychus aequalis

XML Treatment for Sinoxylon unidentatum

XML Treatment for Scobicia chevrieri

XML Treatment for Xyloperthella picea

XML Treatment for Apate monachus
